# MTSS1 and SCAMP1 cooperate to prevent invasion in breast cancer

**DOI:** 10.1038/s41419-018-0364-9

**Published:** 2018-03-01

**Authors:** Jayakumar Vadakekolathu, Shaymaa Ismael Kadhim Al-Juboori, Catherine Johnson, Anne Schneider, Magdalena Elżbieta Buczek, Anna Di Biase, Alan Graham Pockley, Graham Roy Ball, Desmond George Powe, Tarik Regad

**Affiliations:** 1 The John van Geest Cancer Research Centre, School of Science and Technology, Nottingham Trent University, Nottingham, NG11 8NS UK; 2 Department of Cellular Pathology, Queen’s Medical Centre, Nottingham University Hospitals Trust, Nottingham, NG7 2UH UK; 3 Department of Biology, College of science for women, University of Baghdad, Baghdad, Iraq

## Abstract

Cell–cell adhesions constitute the structural “glue” that retains cells together and contributes to tissue organisation and physiological function. The integrity of these structures is regulated by extracellular and intracellular signals and pathways that act on the functional units of cell adhesion such as the cell adhesion molecules/adhesion receptors, the extracellular matrix (ECM) proteins and the cytoplasmic plaque/peripheral membrane proteins. In advanced cancer, these regulatory pathways are dysregulated and lead to cell–cell adhesion disassembly, increased invasion and metastasis. The Metastasis suppressor protein 1 (MTSS1) plays a key role in the maintenance of cell–cell adhesions and its loss correlates with tumour progression in a variety of cancers. However, the mechanisms that regulate its function are not well-known. Using a system biology approach, we unravelled potential interacting partners of MTSS1. We found that the secretory carrier-associated membrane protein 1 (SCAMP1), a molecule involved in post-Golgi recycling pathways and in endosome cell membrane recycling, enhances Mtss1 anti-invasive function in HER2+/ER−/PR− breast cancer, by promoting its protein trafficking leading to elevated levels of RAC1-GTP and increased cell–cell adhesions. This was clinically tested in HER2 breast cancer tissue and shown that loss of MTSS1 and SCAMP1 correlates with reduced disease-specific survival. In summary, we provide evidence of the cooperative roles of MTSS1 and SCAMP1 in preventing HER2+/ER−/PR− breast cancer invasion and we show that the loss of Mtss1 and Scamp1 results in a more aggressive cancer cell phenotype.

## Introduction

Metastasis is a process by which cancer cells that acquired high migratory and invasive properties, leave primary tumours and migrate through the vascular and lymphatic circulatory system to other tissues where they form secondary tumours^1^. This process requires the inactivation of cellular and molecular pathways that maintain cell–cell adhesion and regulate cytoskeleton remodelling and cell motility^2^. In breast cancer 1:5 women have tumours that over express the epidermal growth factor receptor 2 (HER2)/Neu) protein due to amplification of the oncogenic *ERBB2* gene. HER2-positive (HER2+) tumours are among the most aggressive and metastatic^3,4^. The protein Her2 is a member of the epidermal growth factor receptor family. HER2 promotes cell proliferation and survival through the induction of signalling cascades that involve RAS signalling pathways. In this study, we have used a system biology approach to predict RAS interactome pathways from interrogation of a publically available HER2+ breast cancer gene expression array data set. We found that the Metastasis suppressor protein 1 (MTSS1) forms a strong hub of connectivity with other genes that are also significantly expressed in this microarray data set.

MTSS1 belongs to the IMD-family (IRSp53 and MIM (Missing in metastasis) domain) and serves as an actin-binding scaffold protein that is implicated in carcinogenesis and metastasis. It has been proposed that MTSS1 promotes the assembly of actin filaments, and is associated with cytoskeletal organisation and cell motility through elevating RAC1-GTP expression^5–7^. This effect accelerates the kinetics of adherens junction assembly and therefore cell–cell adhesions^7^. MTSS1 is highly expressed in some cancer types and its loss correlates with metastasis and poor prognosis, including breast cancer^8,9^. However, the mechanisms and molecular pathways that regulate the function of MTSS1 are less known. Analysis of the MTSS1 hub of connectivity unravelled several potential interacting partners including the secretory carrier-associated membrane protein 1 (SCAMP1). This molecule belongs to a family of membrane proteins that are involved in post-Golgi recycling pathways and endosome cell membrane recycling^10,11^. The intracellular trafficking of membrane vesicles plays an essential role in the maintenance and the regulation of components of the plasma membrane. Alterations in this cellular pathway can affect cell–cell adhesions and may result in increased cell motility and invasion of cancer cells^12^. On the basis of this background, we hypothesised that the vesicle carrier protein SCAMP1 is involved in stabilising MTSS1 protein trafficking that promotes MTSS1 anti-invasive and anti-metastatic functions by endorsing cell–cell adhesion in HER2+ breast cancer. Moreover, we reveal the dual role of MTSS1 and SCAMP1 in preventing HER2+ breast cancer progression. To better understand the role of MTSS1 and SCAMP1 in tumour progression, we investigated their influence on cell migration and invasion using HER2+ breast cancer cell lines, and MTSS1-expressing and SCAMP1-expressing constructs. Furthermore, we determined the translational importance of this proposal in a clinical setting by showing that loss of MTSS1 and SCAMP1 expression are specifically associated with a worse prognosis in HER2+/ER−/PR− breast cancer. These studies demonstrate that MTSS1, via the carrier protein SCAMP1, prevents cell invasion by promoting cell–cell adhesion via the induction of elevated levels of RAC1-GTP. Collectively, these results support the concept that SCAMP1 promotes MTSS1 protein trafficking that potentiate anti-invasive and anti-metastatic functions. SCAMP1-regulated MTSS1 prevents a more aggressive cancer cell phenotype and its loss is responsible for reduced survival in patients with HER2+/ER−/PR− breast cancer.

## Results

**Fig. 1:**
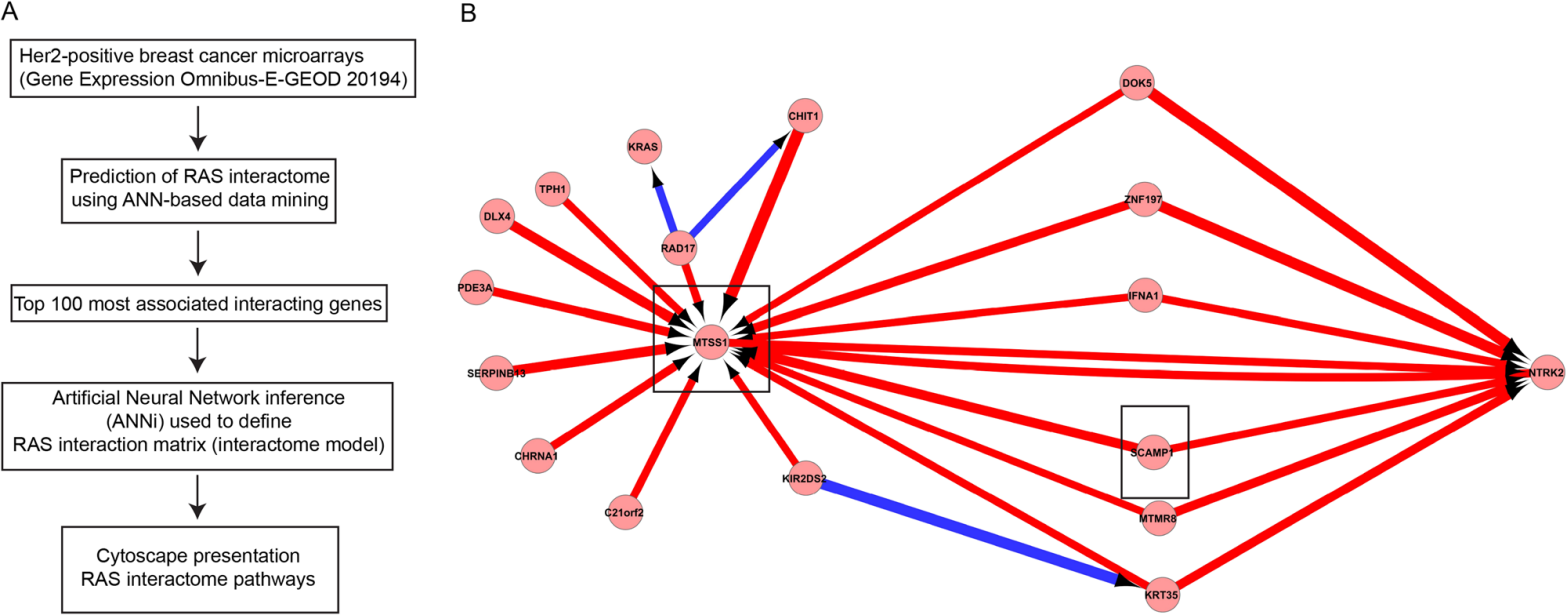
Snapshot of RAS interactome in HER2-positive breast cancer using a system biology approach. **a** Flowchart depicting the multi-tier selection approach for identification of RAS interactome. **b** Pathways of interest are highlighted by rectangles. Red arrows represent a positive regulation and the blues a negative regulation

Identification of MTSS1 and SCAMP1 as key regulators of HER2+ cancer progression by artificial neural network (ANN)-based integrative data mining HER2+ breast cancers are among the most aggressive type of breast cancer. The HER2 receptor is activated upon ligand binding and mediate their influence via RAS signalling pathways that are involved in the regulation of cellular processes including proliferation, survival, and migration. In HER2+ breast cancer, ERBB2 is mutated and results in aberrant activation of RAS signalling that drives HER2+ breast cancer progression. Due to the importance of this pathway, we investigated cellular pathways that may contribute to malignancy progression using a system biology approach. A stepwise ANN method was used to identify an optimised gene signature panel that is associated with the expression of *RAS* in a HER2+ population from the Uppsala breast cancer data set (E-GEOD 20194) (Fig. 1a). The top 100 most associated genes (out of 53,000) were selected for ANN inference (ANNi) that uses a suite of ANN models to study the inter-relationship between markers in a defined set based on the weights of the neural network model^13,14^. Results of the full network inference with filtering to the top 100 interaction values are presented in cytoscape (Fig. 1b and supplementary figure 1). The key hubs (genes with the strongest level of interaction) identified were *MTSS1*, *KRT35*, *NTRK2*, *SLC6A2* and *COL4A4* (Supplementary figure 1). A predominance of strong positive interactions are seen with fewer negative interactions. Interestingly, MTSS1 has previously been shown to play a key role in preventing invasion and metastasis in different types of cancer^9,15–22^, but the mechanisms that regulate its function are unknown. This bioinformatics approach allowed the unravelling of potential interacting partners that may regulate the function of MTSS1 (Fig. 1b).

Among *MTSS1*-predicted interacting partners, we selected two molecules for further studies: SCAMP1 and SERPIN B13. The SCAMP1 is a molecule that is involved in post-Golgi recycling pathways and in endosome cell membrane recycling^10,11^. During these processes, some proteins (e.g., receptors) are a target of proteolysis involving proteinases such as Cathepsins that are involved in endosomal protein catabolism. SERPIN B13 (Hurpin/Headpin) is an intracellular inhibitor of papain-like cysteine proteases (Cathepsins) that has been shown to inhibit specifically Cathepsin K and L^23,24^. We further hypothesised that SCAMP1 may act as a vesicular carrier for MTSS1 and that SERPIN B13 may protect MTSS1 from potential proteolysis during endosome trafficking.

### Clinical significance of MTSS1 and SCAMP1 expression: association with poor prognosis

**Fig. 2:**
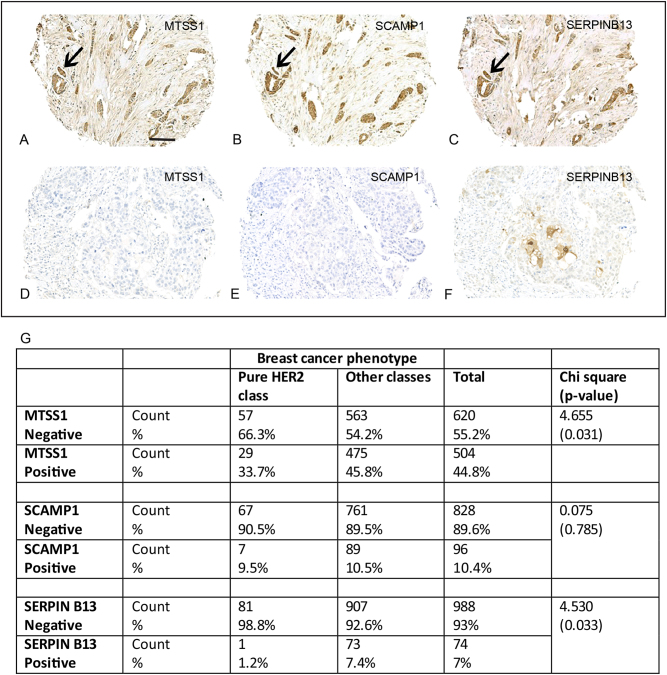
Cytoplasmic staining was detected for (**a**) MTSS1, (**b**) SCAMP1 and (**c**) Serpin B13 protein in the malignant epithelium of breast cancer. Alternatively, the majority of ‘pure’ HER2+ (HER2+/ER−/PR−) tumours showed loss or in (**d**) MTSS1, (**e**) SCAMP1 and (**f**) SERPIN B13 staining. **g** Distribution of MTSS1, SCAMP1 and SERPIN B13 in the ‘pure’ HER2 patient class versus other tumour classes

To investigate whether this hypothesis is translated in the clinical setting, we verified the presence and the expression of MTSS1, SCAMP1 and SERPIN B13 in a cohort of breast cancer patients by immunohistochemistry staining, using specific antibodies to these proteins. When present, cytoplasmic staining was detected in the malignant breast tissue epithelium, with occasional membranous-type staining seen for SCAMP1 and SERPIN B13 (Fig. 2). Expression varied according to tumour molecular phenotype with 73%, 16 and 11% Mtss1 expression seen in oestrogen receptor (ER)-positive, ER-negative and HER2+ patient classes, respectively (*χ*^2^ = 25.84, *p* < 0.0001). Across the whole (unselected) population, MTSS1 expression did not associate with cancer-specific survival (Supplementary figure 2A), but a relationship was detected with MTSS1 loss and metastasis development (*χ*^2^ = 3.83, *p* = 0.05).

**Fig. 3:**
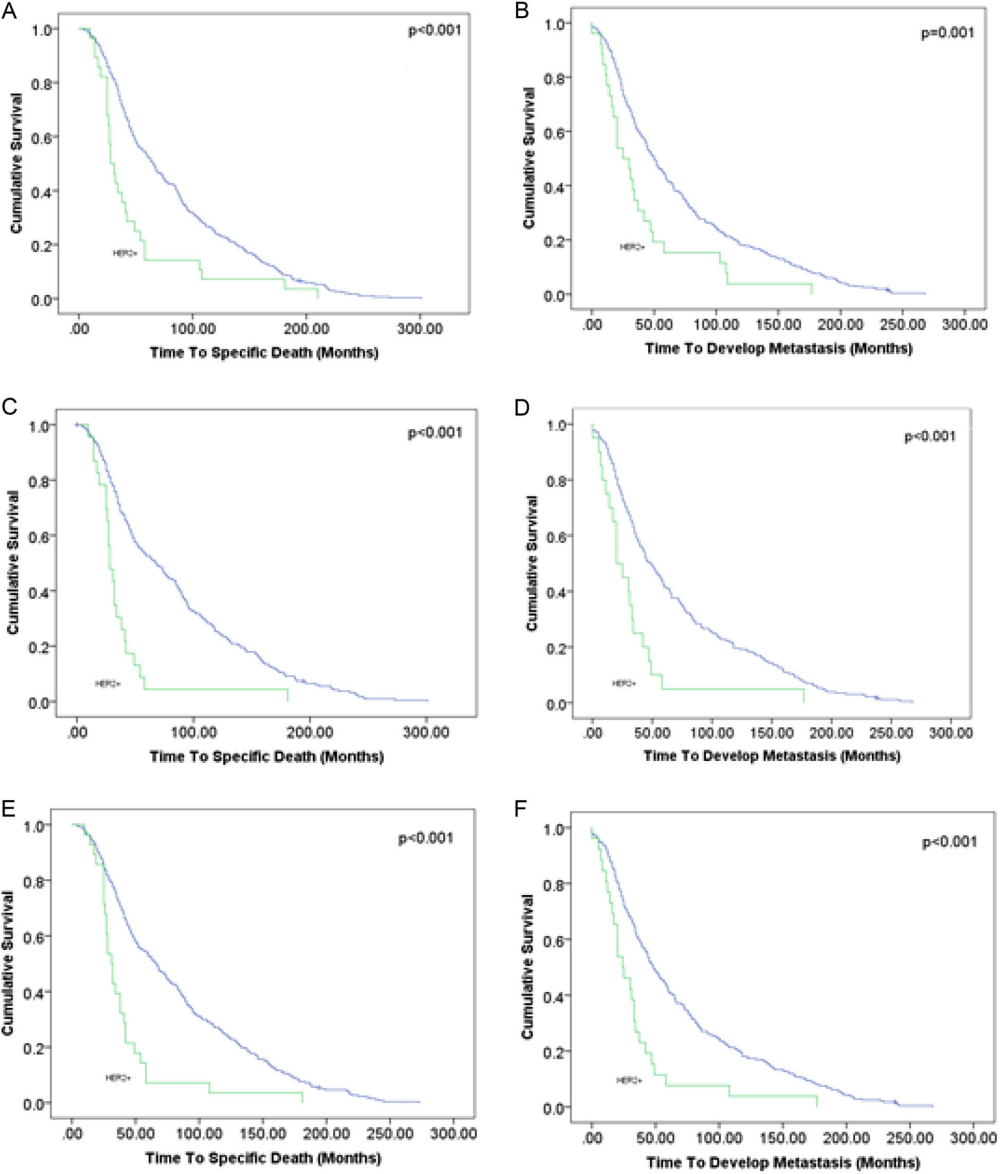
Kaplan–Meier models of survival and metastasis development for MTSS1 (**a**, **b**), SCAMP1 (**c**, **d**) and SERPIN B13 (**e**, **f**) loss, respectively. Less favourable prognosis is seen in patients with ‘pure’ HER2+ tumours compared to other patients showing loss of these biomarkers. Scale bar = 100 μm

Subgroup analysis was performed to test the relationship of MTSS1 expression with disease-specific survival and metastasis outcome in HER2+ breast cancer. Expression of MTSS1 positively correlated with cancer survival (*χ*^2^ = 5.81, *p* = 0.016). Our in vitro studies demonstrated a stronger influence of MTSS1 loss with increasing tumour aggression in cancer cells with a ‘pure’ HER2+ phenotype (HER2+/ER−/PR−). In the clinical samples, 52% of the HER2+ patient class had the HER2+/ER−/PR− phenotype and they differed to their hormonally (ER/PR)-positive counterparts in showing increased loss of MTSS1 (*p* = 0.031; Fig. 2g). HER2+/ER−/PR− phenotype tumours showed increased loss of SCAMP1 protein compared to other HER2+ classes (*p* = 0.034). In addition, a significant positive correlation (*r* = 0.224, *p* = 0.013) between MTSS1 and SCAMP1 expression was identified in the ‘pure’ HER2 group but not in the full patient cohort (Fig. 3).

### Expression of MTSS1 and SCAMP1 prevent cell migration and invasion

**Fig. 4:**
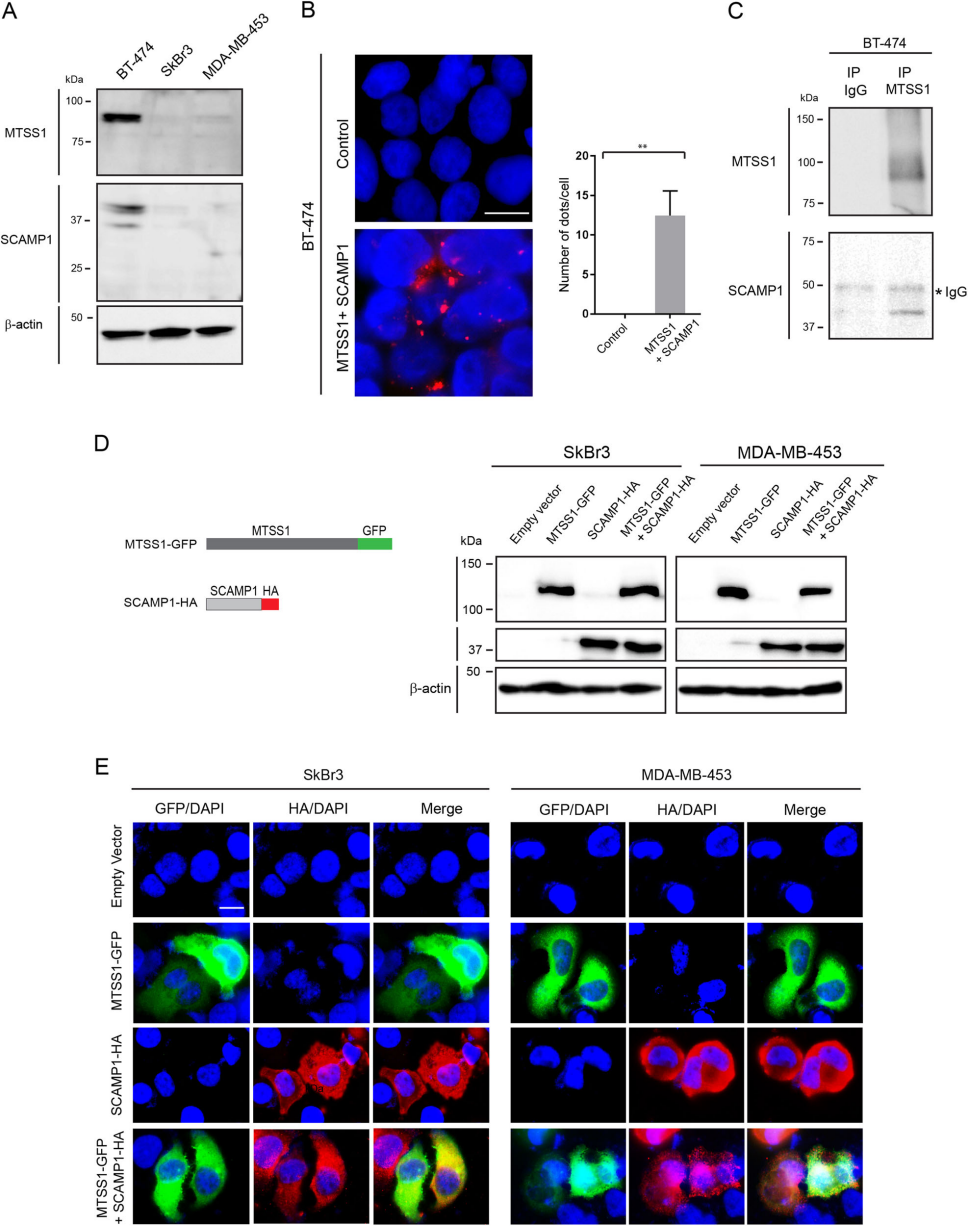
Cellular Interactions between MTSS1 and SCAMP1 in HER2+/ER−/PR− breast cancer cell lines. **a** Protein expression profiles of endogenous MTSS1 and SCAMP1 as assessed by immunoblotting from BT-474, SkBr3 and MDA-MB-453 whole-cell extracts and using MTSS1 and SCAMP1 antibodies. **b** Proximity ligation analysis of endogenous MTSS1 and SCAMP1 interactions in BT-474 cells. Scale bar = 10μm. The graph represents the number of dots/cell. Two tailed *t*-test **p = 0.0024. **c** Interaction of MTSS1 and SCAMP1 as shown by immunoblotting from anti-MTSS1 immunoprecipitates and from BT-474 whole-cell extracts using anti-MTSS1 and anti-SCAMP1 antibodies. **d** Schematic representation of *MTSS1-GFP* and *SCAMP1-HA* mutant constructs and their protein expression profiles as assessed by immunoblotting from SkBr3 and MDA-MB-453 whole-cell extracts and using anti-GFP and anti-HA antibodies. **e** Immunofluorescence images demonstrating expression of MTSS1-GFP and/or SCAMP1-HA in SkBr3 and MDA-MB-453 cells using anti-GFP and anti-HA antibodies. Scale bar = 10 μm

**Fig. 5:**
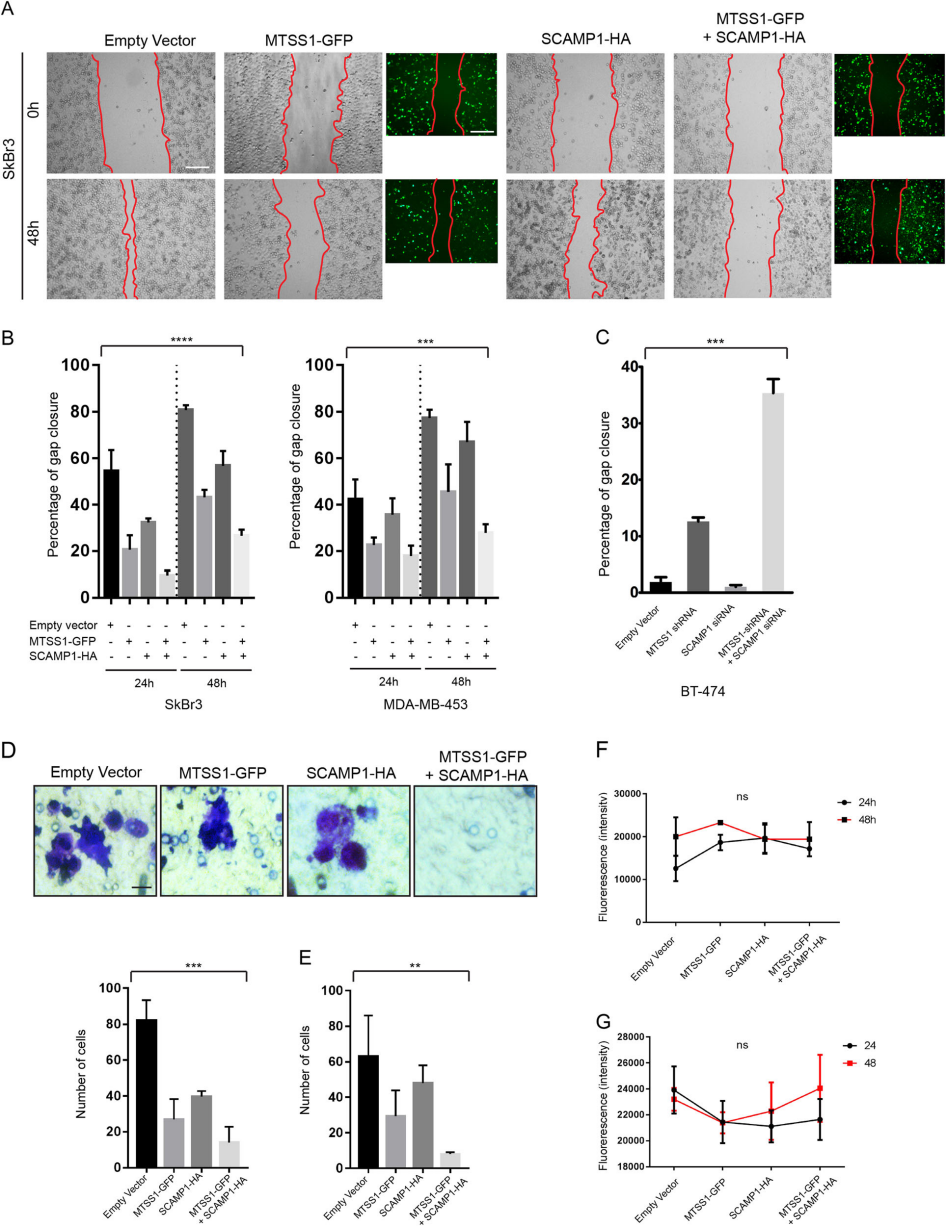
MTSS1 and SCAMP1 expression prevents cell migration and invasion. **a**, **b** Wound-healing assay (Scratch assay) using SkBr3 and MDA-MB-453 expressing *MTSS1-GFP*, *SCAMP1-HA* or both constructs and the corresponding data quantifying gap closure at time points 0, 24 and 48 h following scratching. ANOVA ****P = < 0.0001 and ***P = 0.0001. Scale bar = 200 μm. **c** Wound-healing assay (Scratch assay) using BT-474 cells expressing empty vector, *MTSS1-shRNA*, *SCAMP1 siRNA* or both and the corresponding data quantifying gap closure at time points 24 h following scratching. ANOVA ***P < 0.0001. **d**, **e** Well-cell invasion assay using SkBr3 and MDA-MB-453 cells expressing *MTSS1-GFP*, *SCAMP1-HA* or both constructs and the data relating to the number of invading cells after 48 h of culture. ANOVA ***P = 0.0001 and ANOVA **P = 0.0084. Scale bar = 10 μm. **f**, **g** Graphs representing measurements of cell proliferation of SkBr3 and MDA-MB-453 expressing *MTSS1-GFP*, *SCAMP1-HA* or both constructs (24 and 48 h post transfection). Two-way ANOVA *P*(ns) = 0.1878) and two-way ANOVA *P*(ns) = 0.8040

MTSS1 and SCAMP1 loss is associated with increased invasion that is an early event of the metastatic process and, as seen in the clinical samples, by worsened prognosis. To investigate the expression of endogenous MTSS1 and SCAMP1 in breast cancer cell lines, we performed immunoblotting (IB) using whole-cell extracts from SkBr3 and MDA-MB-453 (HER2+/ER−/PR−) and BT-474 (HER2+/ER+/PR+) human breast cancer cells. Compared to their expression in BT-474 (used as a control), SkBr3 and MDA-MB-453 expressed significantly lower levels of MTSS1 and SCAMP1 (Fig. 4a). This result was consistent with the low expression of MTSS1 and SCAMP1 that were observed in HER2+/ER−/PR− breast cancer tissues (Fig. 2). To determine whether MTSS1 and SCAMP1 interact, we performed a proximity ligation assay (PLA) and immunoprecipitation (IP) experiment using whole-cell extracts from the BT-474 breast cancer cell line. The results from these experiments demonstrated that both proteins interact (Fig. 4b, c).To investigate the role of MTSS1 and SCAMP1 in cell migration and invasion, we generated MTSS1 green fluorescent protein (GFP)-tagged and a SCAMP1 haemagglutinin (HA)-tagged constructs: *MTSS1-GFP and SCAMP1-HA* (Fig. 4a). The constructs were transfected in SkBr3 and MDA-MB-453 cell lines and their protein expression pattern was determined by IB and immunofluorescence (IF) (Fig. 4d, e). To determine the role of MTSS1 and SCAMP1 in cell migration and invasion, we used a wound-healing (Scratch assay) and well-cell invasion assays. SkBr3 and MDA-MB-453 cells expressing MTSS1 or SCAMP1 exhibited a decreased migration (Fig. 5a, b) and invasive (Fig. 5d, e) capacities in comparison to cells expressing the empty vector (Fig. 5). This effect on migration and invasion was more significant when both MTSS1 and SCAMP1 were co-expressed. An increased cell migration of BT-474 breast cancer cells was also observed following combined MTSS1 and SCAMP1 knockdowns, and when compared to the control or when MTSS1 or SCAMP1 were individually knocked down (Fig. 5c and supplementary figure 2B and C). Furthermore, the expression of MTSS1, SCAMP1 or both does not appear to affect the proliferation of transfected cells (Fig. 5f, g). These results demonstrate that MTSS1 and SCAMP1 interact and cooperate in preventing migration and invasion of HER2+/ER−/PR− breast cancer cells.

### MTSS1 and SCAMP1 promote cell–cell adhesion via RAC1-GTP activation in HER2+ breast cancer cell lines

**Fig. 6:**
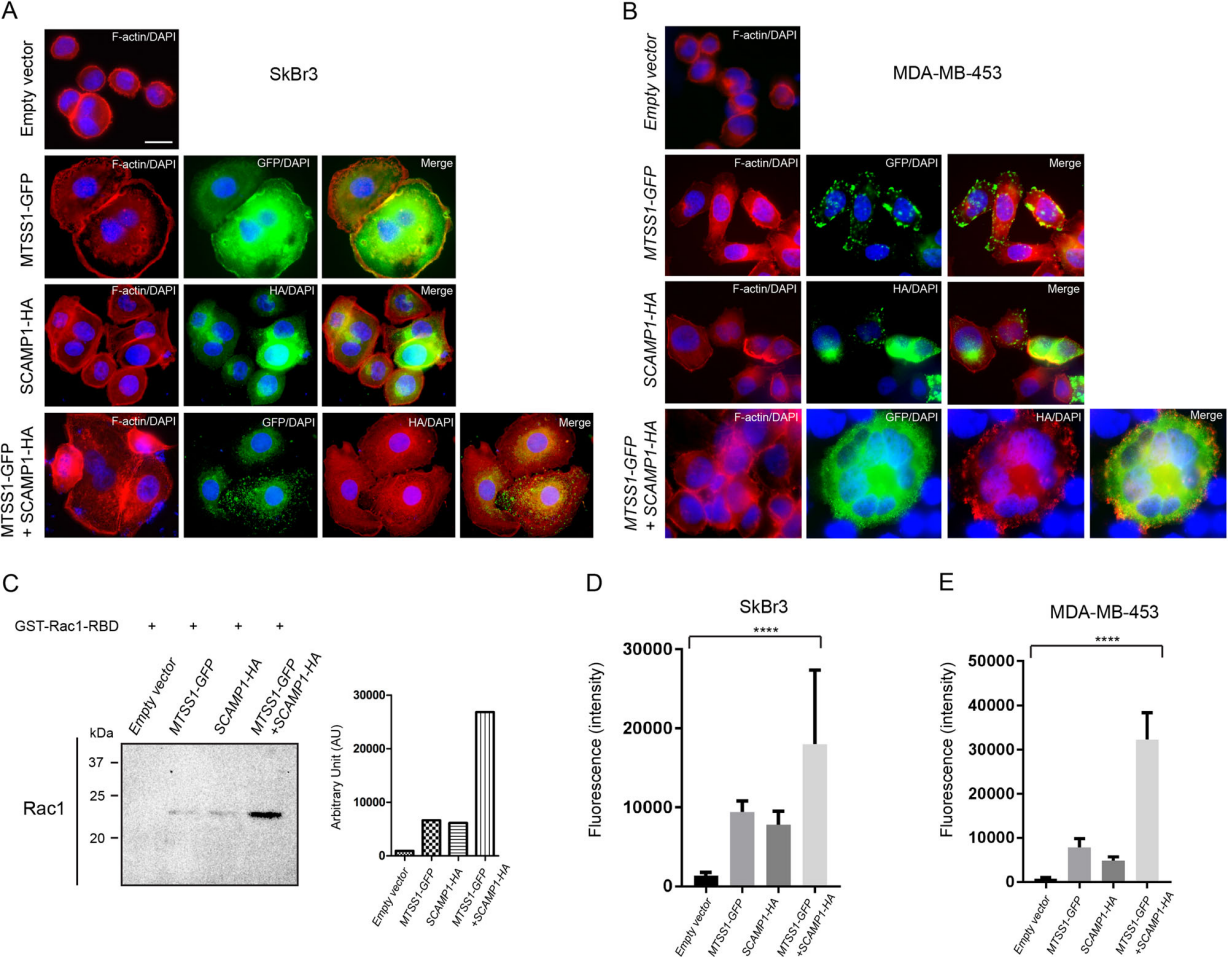
MTSS1 and SCAMP1 expression promotes cell–cell adhesions via promoting the expression of active RAC1. **a**, **b** Immunofluorescence images showing expression of F-actin, MTSS1-GFP and/or SCAMP1-HA in SkBr3 and MDA-MB-453 cells expressing *MTSS1-GFP*, *SCAMP1-HA* or both constructs and using Alexa Fluor® 568 Phalloidin, anti-GFP and anti-HA antibodies. Scale bar = 10 μm. **c** Immunoblotting from GST-PAK1-PBD pull down of RAC1 and from transfected SkBr3 and MDA-MB-453 whole-cell extracts using RAC1− antibody. **d**, **e** Graphs representing measurements from cell adhesion assays performed on SkBr3 and MDA-MB-453 expressing the empty vector, *MTSS1-GFP*, *SCAMP1-HA* or both constructs (48 h post transfection). ANOVA ****P < 0.0001) and ANOVA ****P < 0.0001

The transition from non-invasive to invasive cancer is prevented by cell–cell adhesions that are maintained by collective actions of cellular pathways such as the ones involved in the dynamic reorganisation of the actin cytoskeleton. It has previously been shown that MTSS1 accelerate the kinetics of adherens junction assembly and cell–cell adhesions through elevating RAC1-GTP expression^5–7^. To investigate the mechanism by which MTSS1 and Scamp1 prevent HER2+/ER−/PR− cell invasion, we performed IF staining using Phalloidin (F-actin staining) and antibodies for GFP and HA on cells that were transfected with *MTSS1-GFP and SCAMP1-HA*. Increased cell–cell contacts that are shown by F-actin staining, were observed in SkBr3 and MDA-MB-453 cells expressing MTSS1 or SCAMP1 compared to cells expressing the empty vector (Fig. 6a, b). This increase was significantly important when MTSS1 and SCAMP1 were co-expressed (Fig. 6a, b). To determine whether this event is associated with MTSS1-mediated activation of RAC1-GTP, we used a Glutathione resin-based IP system to pull down active RAC1 (RAC-GTP) from whole-cell extracts that were obtained from SkBr3 cells transfected with the empty vector, *MTSS1-GFP*, *SCAMP1-HA* or a combination of *MTSS1-GFP* and *SCAMP1-HA*. The immunoprecipitates were analysed by IB using an antibody for RAC1. MTSS1-expressing cells showed increased levels of RAC1-GTP expression as previously reported (Fig. 6c)^7^. SCAMP1-expressing cells also showed an increased activity of RAC1-GTP, similar to the one observed with MTSS1-expressing cells. However, co-expression of MTSS1 and SCAMP1 resulted in higher expression level of RAC1-GTP when compared to singularly expressing MTSS1 or SCAMP1 cells. The increased cell–cell contacts in the presence of MTSS1 and SCAMP1 and that were observed by F-actin staining suggests potential increase of cell–cell adhesions. To determine this, we performed a cell adhesion assay using the Vybrant™ Cell Adhesion Assay. Indeed, significant increase in cell–cell adhesions were observed in the presence of MTSS1 and SCAMP1, and when compared to MTSS1 or SCAMP1 alone, and to the control (Fig. 6d, e). Although an increase of cell–cell adhesions were observed in the presence of either MTSS1 or SCAMP1, this increase was significantly lower than the one observed in the presence of expression of both molecules. Taken together, these observations highlight the cooperation of MTSS1 and Scamp1 in preventing HER2+/ER−/PR− cancer progression and provide further insights on early events associated with the pathogenesis of cancer invasion.

## Discussion

Breast cancer is a heterogeneous disease resulting from a highly complex and diverse genomic portrait. Clinical management is determined by clinicopathology assessment, testing for hormonal (ER/PR) receptors and amplification of HER2. Essentially, this results in patient molecular classification that guides treatment regimen selection and survival risk. If improvement in individualised treatment is to be achieved, it is important to gain a better understanding of the cell signalling pathways that govern, or frustrate, patient molecular class membership. Risk of disease-specific death is increased by metastasis development and treatment resistance; the latter may result from interacting or opposing cell signalling pathways, calling for a new combination therapy approach^24^, to reduce metastasis risk. Metastasis involves a multi-step biological process and is characterised by loss of cell:cell adhesion, allowing cancer cells to invade through surrounding tissue^25^.

Cell–cell adhesions are maintained by functional units of multi-protein complexes that are organised into three general classes of proteins; the cell adhesion molecules/adhesion receptors, the extracellular matrix (ECM) proteins, and the cytoplasmic plaque/peripheral membrane proteins^26^. These structures are essential for tissue organisation, homoeostasis and function. The maintenance and regulation of this natural and exceptional machinery rely on a complex network of intracellular and extracellular signals and cellular pathways. During tumour progression, cell–cell adhesions are affected by cytoskeleton alterations, disassembly of cell–cell junctions and consequent changes in cell morphology^27,28^. This enables malignant cancer cells to migrate, invade and metastasis. MTSS1 belongs to the IMD-family (IRSp53 and MIM (Missing in metastasis) domain) that act as cytoskeletal scaffold proteins and regulate cytoskeletal dynamics through interaction with Rac1, actin and actin-associated proteins^5–7,19,29^. MTSS1 is highly expressed in primary tumours and its loss has been found to correlate with metastasis and poor prognosis in different types of cancer^9,15–22^. However, other studies have shown that MTSS1 expression may be associated with increased invasion and metastasis in subtypes of malignant melanoma, non-small cell lung (NSCLC) and breast cancer^30–32^. These observations indicate differences in functionality for MTSS1 according to tumour specificity. Although MTSS1 was expressed in some HER2+ molecular subtypes, we observed its expression was markedly decreased in clinical tumour samples with the HER2+/ER−/PR− phenotype, suggesting hormonal androgen expression may mitigate for the loss of MTSS1 tumour suppressor functionality. This decrease of MTSS1 expression correlated with reduced survival and worse prognosis in HER2+/ER−/PR− breast cancer patients. Furthermore, this clinical finding was validated in our in vitro invasive models of breast cancer. We showed that restoring Mtss1 expression in HER2+/ER−/PR− breast cancer cell lines promoted cell–cell adhesion and prevented cell invasion. These events were associated with the capacity of MTSS1 to induce elevated levels of RAC1-GTP, as previously reported^7^. Our clinical findings support a recent study showing decreased survival in trastuzumab-treated breast cancer patients with a HER2/ER− tumour profile compared to HER2/ER+ (HER2/luminal B) patients^33^. The current findings provide a possible explanation for the difference seen in the two patient groups.

Although we have some understanding of the role of MTSS1 in cancer, cellular pathways that may regulate its function are not well-known. Using a system biology approach, we identified several potential interacting partners, of which SCAMP1 was selected for further studies. This selection was based on the role of this molecule in post-Golgi recycling pathways and in endosome cell membrane recycling^10,11^. MTSS1 transport to the cell surface may be facilitated by SCAMP1. Moreover, the role of SCAMP1 in cancer is still unknown. Indeed, we found that SCAMP1 expression correlated with Mtss1 expression in tumours with the HER2+/ER−/PR− phenotype and its expression was decreased similarly to MTSS1 in the HER2+/ER−/PR− breast cancer class. Likewise, restoring SCAMP1 expression in HER2+/ER−/PR− breast cancer cell lines also promoted cell–cell adhesion and prevented cell invasion. Importantly, co-expression of MTSS1 and SCAMP1 resulted in a more efficient inhibition of cell invasion and increased cell–cell adhesion in our cancer models when compared to the expression of Mtss1 or Scamp1 alone. Taken together, these results confirm our hypothesis of synergistic interaction between MTSS1-mediated and SCAMP1-mediated cellular pathways in preventing HER2+/ER−/PR− breast cancer invasion. Our findings lead us to propose that further investigations are needed to assess the relationship between loss of MTSS1 in HER2 patients and its relationship to trastuzumab response.

## Materials and methods

### Antibodies

For this study, we used antibodies to MTSS1 (1:30 for IHC, LS-B1818, LifeSpan BioSciences, Seattle, WA, USA), MTSS1 (1:250 for IB, 4386, Cell Signaling Technology, Danvers, MA, USA), MTSS1 (1:50 for PLA, sc-101390, Santa Cruz Biotechnology, Dallas, TX, USA), SCAMP1 (1:25 for IHC, 1:100 for PLA, 15327-1-AP, Proteintech, Rosemont, IL, USA), SERPIN B13 (1:125 for IHC, NBP2-01312, Novus Biological, Littleton, CO 80120, USA), GFP (1:1000, ab1218, Abcam, Cambridge, UK), HA (1:1000 for IB, 1:200 for IF, A190-108 A, Bethyl Laboratories Inc., Montgomery, TX, USA), β-actin (1:5000 for IB, A5441, Sigma-Aldrich, St Louis, MO, USA).

### Plasmids and constructs

*MTSS1-GFP* and *SCAMP1-HA* fusion genes were synthesised using custom Gene Synthesis Service (GenScript, Piscataway, NJ, USA) and incorporated into PUC57Kan cloning vector. The fusions genes PUC57Kan vector were further sub-cloned into PCDNA 3.1(+) expression vector (Invitrogen, ThermoFisher scientific) using 5′-*Bam*H1 and 3′-*Xba* I restriction sites. *MTSS1-shRNA* lentiviral constructs were purchased from Sigma-Aldrich (SHCLNG-NM_014751) and were used as previously described^34^. SCAMP1 siRNAs were purchased from QIAGEN (FlexiTube GeneSolution GS9522 for SCAMP1) and were used following manufacturer recommendations.

### Cell lines, growth conditions and cell transfection

SkBr3 breast cancer cells (HTB-20, ATCC) were cultured in Lonza/12–168 McCoy’s media with 10% fetal bovine serum (FBS). MDA-MB-453 cells (ACC65, Leibniz Institute DSMZ-German Collection of Microorganisms and Cell Cultures) were cultured in 90% Leibovitz’s L-15 medium with 10% FBS. BT-474 cells (HTB-20, ATCC) were cultured in ATCC/46-XHBRI-CARE media with 10% FBS and 1.5 g/L Sodium Bicarbonate. Plasmids were transfected using Lipofectamine™ 3000 Reagent (L3000001, ThermoFisher Scientific) and following manufacturer recommendations.

### Cell migration, invasion, proliferation and adhesion assays

For wound-healing assay (Scratch assay), SkBr3 and MDA-MB-453 cells expressing the empty vector, *MTSS1-GFP*, *SCAMP1-HA* or both were cultured to 80% confluence and serum starved for 24 h, after which a scratch was made in the middle of each well using a 10 μl pipette tip. Images from triplicate experiments at 0, 24 and 48 h were taken and the distances between the edges of the scratch were measured at 3 different points using Carl Zeiss AxioVision software^35^ (Buczek et al., 2016). The measurements were expressed as percentages of gap closure. For the well-cell invasion assay, we used the colorimetric QCM ECMatrix Cell Invasion Assay (ECM550, Millipore, Ltd.) and the cell count of invasive cancer cells were performed following the manufacturer’s recommendations. For the proliferation assay, cells were cultured (10000 cells per/well) and then transfected for 24 h and 48 h. Media were removed and the proliferation was assessed using the CyQUANT® NF Cell Proliferation Assay Kit (C35007, Life Technologies, Ltd.) and following the manufacturer’s recommendations. Cell–cell adhesions were assessed following transfections (48 h) and using Vybrant™ Cell Adhesion Assay Kit (V13181, Fisher Scientific), and the manufacturer’s recommendations.

### IB and IP

The experimental procedures were performed as described previously^34^. For IB, the cells lysed in 1× solution containing 50 mM Tris-HCl (pH 6.8), 100 mM dithiothreitol, 2% (w/v) SDS, 0.1% (w/v) bromophenol blue, and 10% (v/v) glycerol, and loaded onto Tris/glycine SDS–polyacrylamide gels for electrophoresis. The proteins were transferred onto Amersham Hybond-P PVDF membranes (GE Healthcare). Membranes were blocked with 10% (w/v) Marvel milk powder/phosphate-buffered saline (PBS) solution with 0.1% (v/v) Tween-20 (PBST). Membranes were washed in PBST, membranes were incubated with primary antibodies (in blocking solution) overnight at 4 °C followed by washing and incubation with secondary antibodies for 1 h at room temperature prior to visualisation using Clarity™ Western ECL Substrate (Bio-Rad Laboratories) and a CCD camera (SynGene). For IP, the cells were lysed in IP buffer (0.05 M Tris pH 7.4, 0.15 M NaCl, 0.5% (v/v) Triton X-100, and 0.001 M EDTA). The extracts were pre-cleared for 1 h at 4 °C using Protein G Plus-Agarose beads (IP08, Calbiochem) and corresponding IgG. GFP antibody was incubated with agarose beads in IP buffer for 2 h before adding the pre-cleared extracts for overnight IP. The beads were washed 4 times in IP buffer and suspended in lysis buffer, after which samples were analysed by IB. For active Rac1 detection, we used the Active Rac1 Detection Kit and following manufacturer recommendations (#8815, Cell Signaling Technology).

## Bioinformatics analysis

### Monte Carlo Cross validation

Prior to ANN training, the data was randomly divided into three subsets; 60% for training, 20% for testing (to assess model performance during the training process) and 20% for validation (to independently test the model on data completely blind to the model). This process of random sample cross validation also contributed to the reduction of over fitting to the data.

### Architecture

The ANN modelling undertaken used a supervised learning approach applied to a three-layer multi-layer perceptron architecture. The initial weights matrix was randomised with a standard deviation of 0.1 to reduce the risk of over fitting the data. The ANN architecture was initially constrained to two hidden nodes in the hidden layer also for this reason. Hidden nodes and the output node incorporated a sigmoidal transfer function. During training, weights were updated by a feed-forward back propagation algorithm^36^. Learning rate and momentum were set at 0.1 and 0.5, respectively. The output node was coded as 0 if the patient showed low expression of RAS, and 1 if the patient showed high expression of RAS.

### Network inference analysis

Network inference analysis was undertaken on the top 100 probes from step 1 of the classifier development. Thus the single non-orthogonal probes were analysed. This approach^13,14^ uses a suite of ANN models to study the inter-relationship between markers in a defined set. In the algorithm all markers are used to predict 1 marker and the weights of the model analysed. This is then repeated for each marker in turn so that in the process all markers are used as outputs and a matrix of interactions identified. This matrix is then filtered to identify the strongest interactions and the most connected/influential genes. The resulting interactions were presented using Cytoscape.

### Immunohistochemistry

The relationship between MTSS1 to its carrier molecule, SCAMP1, and its stabilisation protein, SERPIN B13 was investigated in a cohort of wax embedded tissue microarrayed (TMA) breast cancer using immunohistochemistry. The TMA comprises tumour samples obtained from unselected patients diagnosed with primary operable breast cancer (TNM stage I-IIIa) that presented at the Nottingham Breast Unit between 1988 and 1998. Prospectively collected clinical and pathology data is available including development of recurrence and distant metastases (DM), survival time and disease-free interval (DFI). Breast cancer-specific survival (BCSS) was defined as the time (in months) from the date of the primary surgical treatment to the time of death from breast cancer. DFI was defined as the interval (in months) from the date of the primary surgical treatment to the first locoregional or distant metastasis. Patient management and treatment regimens have been previously described^1^. Protein expression was immunohistochemically assessed using a Ventana Benchmark Ultra platform (Roche Group, Arizona, US). Malignant epithelium was microscopically dichotomously scored for levels of staining intensity where 0: negative or low intensity staining, and 1: moderate to strong intensity. REMARK guidelines^37^ were used in the whole patient series and the number of scorable patients was found to be less than those originally arrayed due to tissue detachment and absence of malignant tissue. A Pearson's *χ*^2^-test with cross-tabulation tables was performed to assess associations between the biomarkers and clinical survival data, using SPSS (Version 21; IBM, US). Kaplan–Meier plots with log-rank tests were used to model biomarker associations for disease-specific survival (DSS) and time to metastasis development. *χ*^2^-tests were performed on the whole (unselected) patient cohort, followed by subset analysis of HER2 (cerbB2)-positive patients, and Pearson's correlation tests were used to assess the association between different antigen biomarkers with clinical outcome. HER2+ patients were stratified according to hormonal oestrogen and progesterone receptor status.

### IF staining and PLA

The IF staining was performed as previously described^34^. Cells were fixed in 4% (w/v) paraformaldehyde and then treated as follows: the sections or cancer cells were washed three times in 1× PBS for 10 min each, blocked and permeabilised in 10% (w/v) bovine serum albumin in 0.1% (v/v) PBS-Tween, incubated overnight with primary antibody (in blocking solution), washed three times for 10 min each with 1× PBS, incubated for 1 h with secondary antibody (in blocking solution) and washed three times with 1× PBS. Sections and melanoma cells were counterstained and mounted with DAPI fluorescent medium (Vector Laboratories) for IF microscopy. The PLA assay was performed using the Duolink^®^ In Situ Red Starter Kit Mouse/Rabbit (DUO92101, Sigma-Aldrich, St Louis, MO, USA) and following the manufacturer recommendations. The number of dots/cell was determined by counting the number of dots from three independent images.

## Electronic supplementary material

Supplementary figure 1

Supplementary figure 2

Supplementary figure Legends
