# Correction: MTSS1 and SCAMP1 cooperate to prevent invasion in breast cancer

**DOI:** 10.1038/s41419-020-2401-8

**Published:** 2020-03-23

**Authors:** Jayakumar Vadakekolathu, Shaymaa Ismael Kadhim Al-Juboori, Catherine Johnson, Anne Schneider, Magdalena Elżbieta Buczek, Anna Di Biase, Alan Graham Pockley, Graham Roy Ball, Desmond George Powe, Tarik Regad

**Affiliations:** 1 The John van Geest Cancer Research Centre, School of Science and Technology, Nottingham Trent University, Nottingham, NG11 8NS UK; 2 Department of Cellular Pathology, Queen’s Medical Centre, Nottingham University Hospitals Trust, Nottingham, NG7 2UH UK; 3 Department of Biology, College of science for women, University of Baghdad, Baghdad, Iraq

**Correction to: Cell Death and Disease**

10.1038/s41419-018-0364-9 published online 1 March 2018

The financial support for this Article was not fully acknowledged. The Acknowledgements should have included the following:

“This study was supported by the European Union’s Horizon 2020 research and innovation program under the Marie Sklodowska-Curie grant agreement no 641549, Immutrain.”

The PDF and HTML versions of the paper have been modified accordingly.

