# Supplementary figures and images for: MTSS1 and SCAMP1 cooperate to prevent invasion in breast cancer
Source: Cell Death Dis. 2018 Mar 1;9(3):344. doi: 10.1038/s41419-018-0364-9 (PMC5832821; doi:10.1038/s41419-018-0364-9)

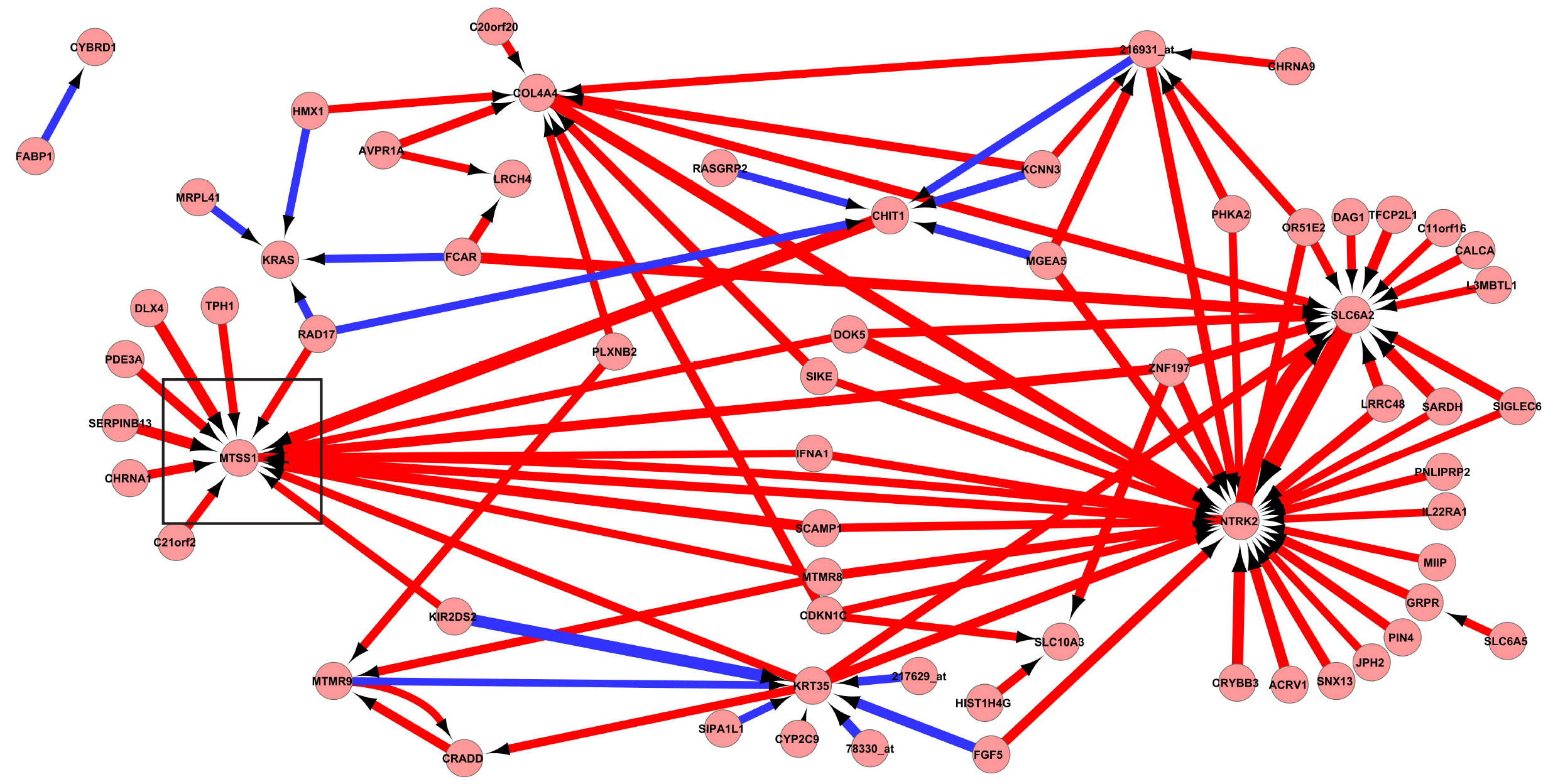

Supplement: Supplementary file 1 — Supplementary figure 1 [file 41419_2018_364_MOESM1_ESM.jpg]

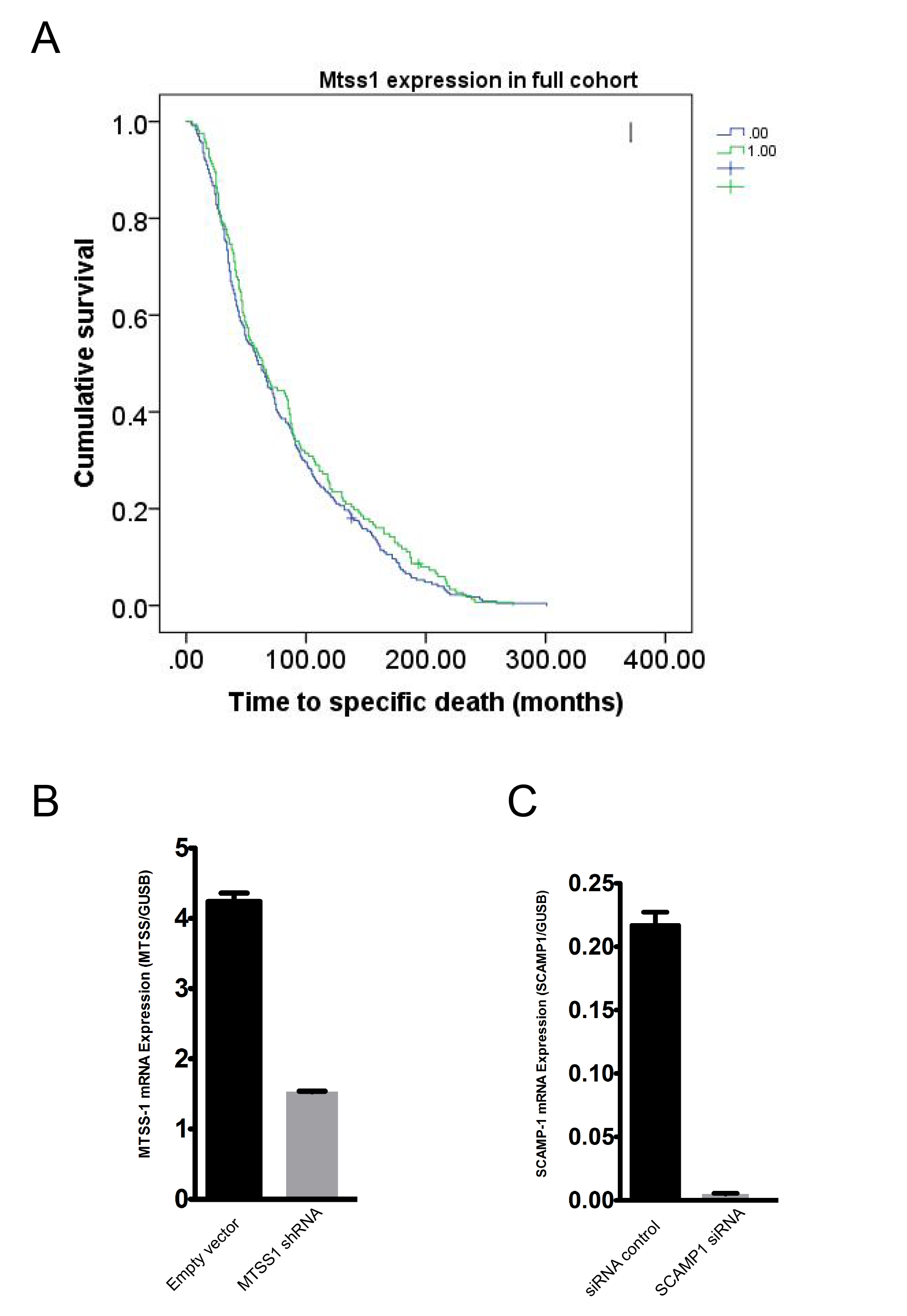

Supplement: Supplementary file 2 — Supplementary figure 2 [file 41419_2018_364_MOESM2_ESM.jpg]
