# Supplementary material for: MTSS1 and SCAMP1 cooperate to prevent invasion in breast cancer
Source: Cell Death Dis. 2018 Mar 1;9(3):344. doi: 10.1038/s41419-018-0364-9 (PMC5832821; doi:10.1038/s41419-018-0364-9)
Supplement: Supplementary file 3 — Supplementary figure Legends [file 41419_2018_364_MOESM3_ESM.docx]

**Supplementary figure Legends**

**Supplementary figure 1:** RAS interactome in HER2-positive breast cancer using a system biology approach. Pathways of interest are highlighted by rectangles. Red arrows represent a positive regulation and the blues a negative regulation.

**Supplementary figure 2:** (A) Kaplan-Meier survival plot for high/low MTSS1 protein expression in full patient cohort. (B) (C) qRT-PCR of *MTSS1* and *SCAMP1* mRNA expression in BT-474 control vs. MTSS1 shRNA, and following SCAMP1 knockdown with siRNA.
